# A randomized, parallel control and multicenter clinical trial of evidence-based traditional Chinese medicine massage treatment VS External Diclofenac Diethylamine Emulgel for the treatment of knee osteoarthritis

**DOI:** 10.1186/s13063-022-06388-5

**Published:** 2022-07-08

**Authors:** Wang Wen-yue, Xu Ying-peng, Ding Quan-mao, Xie Li-min, Wang De-zhi, Bai Yang, Wang Li-su, Li Yu-bin, Niu Zhi-jun, Ma Yan-xu, Chen Wu-zhong, Bai Li-qun, Liu Yang, Jin Li-kun

**Affiliations:** 1 Guang’anmen Hospital, China Academy of Chinese Medical Sciences, Beijing, China; 2 Beijing Xuan Wu TCM Hospital, Beijing, China; 3 Beijing’s Capital Medical University Traditional Chinese Medicine Hospital, Beijing, China; 4 Dongfang Hospital of Beijing University of Chinese Medicine, Beijing, China; 5 Beijing Fengsheng Trauma-Orthopedic Hospital of Traditional Chinese Medicine, Beijing, China

**Keywords:** Clinical effect, Safety evaluation, Evidence-based traditional Chinese medicine massage, External Diclofenac Diethylamine Emulgel, Knee Osteoarthritis, Magnetic resonance imaging, Multi-center, Clinical trial

## Abstract

**Background:**

Both massage and topically administered NSAIDs are safe and effective treatments for knee osteoarthritis (KOA); however, different massage technique sects in China caused assessment difficulties for the treatment of KOA. In order to standardize the massage techniques and procedures, we organized multi-disciplinary experts in China to acquire an evidence-based traditional Chinese medicine massage treatment of knee osteoarthritis. The purposes of this study will be to provide clinicians a complementary and alternative therapy for patients and to evaluate the efficacy and safety of evidence-based traditional Chinese medicine massage treatment of KOA compared to External Diclofenac Diethylamine Emulgel.

**Methods and design:**

A randomized controlled trial in which 300 participants diagnosed with KOA will be recruited and randomly allocated to either the experimental group or the control group in a ratio of 2:1. Two hundred participants will receive evidence-based traditional Chinese medicine massage 2 sessions per week for 10 weeks as the experimental group, and 100 participants will receive External Diclofenac Diethylamine Emulgel 3–4 times per day for 10 weeks as the control group. The patients in the two groups will receive follow-up at two time points at 5 weeks and 10 weeks from the beginning of treatment, respectively. The MRI scans and X-ray will be performed at baseline and at the end of the intervention. The primary outcome will be the changes in the Western Ontario and McMaster Osteoarthritis Index (WOMAC). Secondary outcomes will be measured by the PRO scale for knee osteoarthritis based on the concept of traditional Chinese medicine (Chinese scale for knee osteoarthritis (CSKO)), X-ray evaluation, and MRI scan evaluation. The data of WOMAC and CSKO will be analyzed at the baseline, 5 weeks, and 10 weeks from the beginning of treatment. The data from MRI scans and X-rays will be analyzed at baseline and at the end of the intervention. The significance level sets as 5%. The safety of interventions will be evaluated after each treatment session.

**Discussion:**

This study will provide clinicians with much-needed knowledge for the treatment of KOA through a controlled trial.

**Trial registration:**

Chinese Clinical Trial Registry ChiCTR1800014400. Registered on 10 January 2018

## Introduction

Knee osteoarthritis (KOA) ranks highly among global causes of disability and chronic pain, particularly in people over 50 years of age [1]. Structured exercise programs, dietary weight management, and mind-body exercise (such as tai chi and yoga) were considered by the panel to be effective and safe for all patients with knee OA [2–5]. These updated OARSI guidelines recommended non-pharmacological as the core treatments for KOA in all cases [6].

Massage is a safe and non-pharmacological treatment with limited contraindications and no known serious adverse events [7]. Traditional Chinese massage has a positive effect on KOA and widely used in China. In recent several studies have shown that Chinese massage therapy decreased pain and may improve extensor muscle strength, and it may improve walking ability for these KOA patients; however, different massage sects in China caused assessment difficulties for the treatment of KOA [8, 9]. In order to standardize massage techniques and procedures, we organized multi-disciplinary experts in China to conduct a questionnaire survey and field discussions and successfully acquire an evidence-based traditional Chinese medicine massage treatment of knee osteoarthritis [10].

Topically administered non-steroidal anti-inflammatory drugs (NSAIDs) such as Diclofenac solution attained significantly greater improvement in pain reduction in patients with KOA and are widely used in the world [11–14]. However, local skin reactions happened more commonly in patients treated with topical NSAIDs [15].

In order to provide clinicians a complementary and alternative therapy for patients with KOA, and to determine whether evidence-based traditional Chinese medicine massage is a safe and effective therapy, we design and conduct the trial. Compared to topical NSAIDs (e.g., Diclofenac Emugel), which is strongly recommended (level 1a), massage is conditionally (level 3) recommended by the OARSI guidelines [6]. In the trial, we hypothesized that the evidence-based traditional Chinese medicine massage group would be non-inferior to the External Diclofenac Diethylamine Emulgel group in the treatment for KOA. In order to enhance the reliability of evidence-based traditional Chinese medicine massage in the treatment of KOA, we take the External Diclofenac Diethylamine Emulgel as the control group to evaluate the efficacy and safety in the study. In the overall design of the experiment, this scheme has randomization, control, and multi-center clinical trial.

## Methods and design

**Fig. 1:**
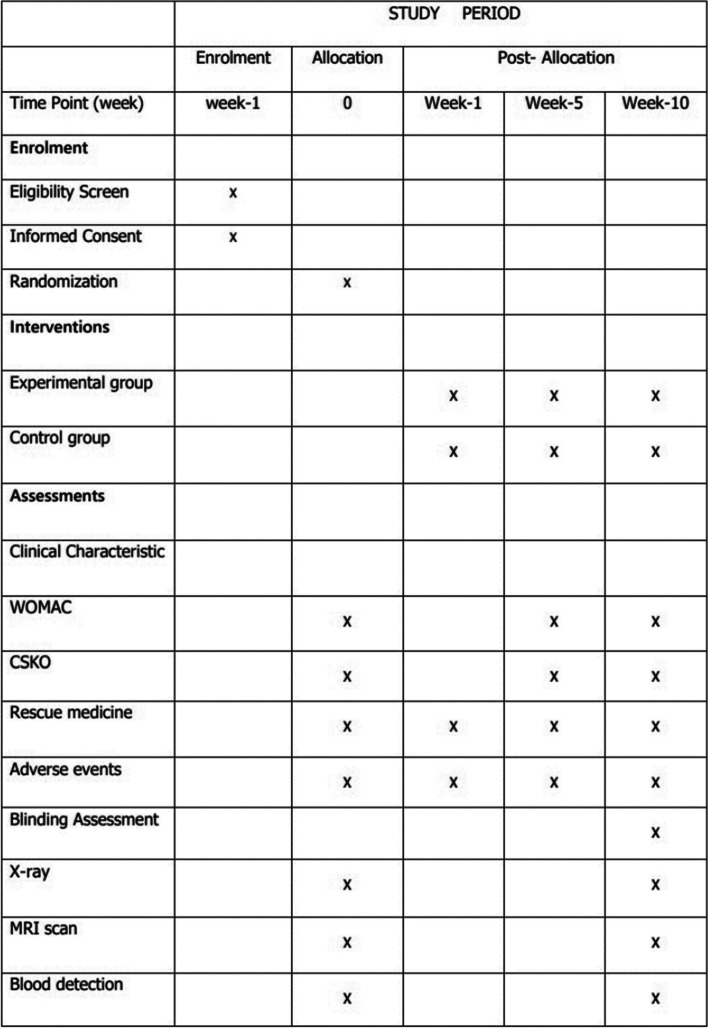
Schedule of enrollment,intervention, and assessment

The main purpose of this study is to compare the safety and efficacy of evidence-based traditional Chinese medicine massage and External Diclofenac Diethylamine Emulgel in KOA patients. Three hundred participants will be recruited for the study. Clinical data measurements, X-rays, and MRI scans will be evaluated at baseline and the end of treatment (Fig. 1). The study has been approved by the ethical committees of Guang’anmen Hospital (NO:201-135-KY-1) and registered in the Chinese Clinical Trial Registry (http://www.chictr.org.cn/showproj.aspx?proj=24457). The registration number is ChiCTR1800014400. The protocol will be reported following Standard Protocol Items: Recommendations for Interventional Trials (SPIRIT) statement.

### Study setting and recruitment

The study will be carried out at the five hospitals: Guang’anmen Hospital, Beijing’s Capital Medical University Traditional Chinese Medicine Hospital, Dongfang Hospital of Beijing University of Chinese Medicine, Xuan Wu TCM Hospital Beijing, and Beijing Fengsheng Trauma-Orthopedic Hospital of Traditional Chinese Medicine. The study will recruit participants with KOA who meet the diagnostic criteria of the American College of Rheumatology through hospital outpatient [16]. WeChat official account is one of the Chinese popular social media platforms of Guang’anmen Hospital and brochures. Meanwhile, the recruitment of participants has started in June 2018.

### Participants

#### Inclusion criteria

The following are the inclusion criteria:

1. Meet the diagnostic criteria of knee osteoarthritis according to the American College of Rheumatology (ACR) criteria [16].
2. Patients who have WOMAC pain scores of 25 or more out of 100 will be included.
3. K-L classification ≥ 1 [17].
4. Age 40–80 years, male or female.
5. Signed informed consent.
6. If the patient has two affected knees of which only one meets the ACR criterion and Kellgren-Lawrence grade II or III [16], only this knee will be evaluated. In the case that both knees are affected in accordance with the inclusion criteria (ACR and Kellgren-Lawrence grade II or III), the more painful knee will be chosen for evaluation.

#### Excluded cases criteria

The following are the exclusion criteria:

1. Patients with secondary osteoarthritis
2. Patients with obvious unstable knee joint (ACL, PCL, MCL injury) [18–20]
3. Patients who have received intra-articular injection of sodium hyaluronate or hormone
4. List excluding comorbidities: severe osteoporosis, diabetes, lower extremity vascular disease, limb malformation or nerve system disease, severe mental illness, unable to understand others’ intentions or express themselves correctly
5. Pregnant women and those trying to conceive or currently breastfeeding
6. Patients who have combined heart, lung, brain, liver, kidney, and hematopoietic system and other serious diseases
7. Patients who have a contraindication for MRI
8. Patients who have an allergy history for investigational products and have an allergic constitution
9. Patients who are participating in other clinical trials
10. Patients who have been used to alcoholic and/or psychoactive drug substances, drug abuser, and relier
11. According to the researchers’ judgment, other diseases or conditions that may reduce the possibility or complicate the inclusion, such as frequent changes in the work environment and unstable living environment, which may easily lead to loss of follow-up

#### Dropout criteria

Participants who do not complete the clinical protocol for the following reasons should be considered as dropped out:

1. The patient quits (poor efficacy or adverse reactions).
2. Loss to follow-up.
3. Researchers remove the patient (poor compliance or serious adverse events).

#### Comprehensive suspension criteria

The trial will be suspended if:

1. The investigators discover a significant safety problem.
2. The therapeutic effect is poor (we will assess the therapeutic effect in week 5).
3. There is a major mistake in the plan.
4. The sponsor has a huge problem in funding or management.

### Informed consent and participant safety

Participants will be required to sign an informed consent and have the same chance to receive appropriate treatment. The safety of participants will be monitored in every visit. Two attending physicians and two responsible joint care specialists will be responsible for the collaboration and guidance of clinicians during each clinical trial and implementation. The two chief joint surgeons constitute the endpoints Adjudication Committee, which is responsible for the overall supervision of the clinical trials.

During the treatment, any adverse events, such as local skin dryness, skin irritation, fracture, dislocation, or increased pain should be recorded and reported to the relevant responsible unit and the ethics committee in time. If the adverse events such as death, fractures, and organs injury are confirmed to be related to the study, the Ethics Committee of Guang’anmen Hospital, China Academy of Chinese Medical Sciences, has the right to discontinue or modify allocated interventions. If an adverse event occurs during the clinical trial, medical experts will be engaged to assess and investigate the actual cause, and medical and financial compensation will be made to participants. If the participants cannot tolerate these adverse reactions, treatment will be discontinued and the participants will be withdrawn from the study.

### Randomization

In this study, this clinical research adopts randomized blocks, and cases will be randomly assigned to each center. The results of the random groups will be placed in sealed envelopes, which are numbered consecutively. When the patients are enrolled, they take the envelopes in the order of enrollment and will be grouped according to the contents of the envelopes. In the waiting list group, in a ratio of 2:1, there are 200 cases in the experimental group and 100 cases in the control group. The random sequence will be generated by an independent professional statistician who is not involved in the study with the SPSS software (SPSS 20.0, SPSS Inc., Chicago, IL, USA). The random numbers will be stored by a fixed non-involved person.

### Blinding

Due to the operation characteristics of massage therapists, the massage therapists and the participants cannot be blinded who will be informed of the grouping of participants before treatment; however, massage therapists will be blinded to pain and function scores, as well as baseline imaging results. Participants will be told that they will randomly receive one of the interventions after enrollment. The outcome evaluators and data statisticians will be blinded to the group allocation, and their responsibility is to collect and analyze the data.

### Sample size

According to the literature, the average effective rate of treating knee osteoarthritis only through massage is 95.6% and 79.8% by application of external diclofenac diethylamine emulsion latex [15]. According to the sample size calculation formula, the minimum sample size of each group is *n* = 85.49. Considering expulsion case and getting significant results, at the same time, in order to implement layered research in the experimental group, we planned the ratio of the cases in the experimental group and the control group is 2:1, and the number of included cases was 300. There were 200 cases in the experimental group, and 100 cases were in the control group.

### Interventions

#### Experimental group

Two hundred patients in the experimental group will receive 20 sessions of manual massage treatment (2 sessions weekly for 10 weeks,10 min one session). Use the definite evidence-based traditional Chinese medicine massage technique for knee osteoarthritis to do the massage treatment [10].

During the treatment period, other Chinese and Western drugs and treatment methods (including physical therapy and psychotherapy, etc.) for knee osteoarthritis are not used. In cases of intolerable knee pain, the patients will be instructed to take loxoprofen sodium tablets (60 mg, oral 3/day) as a rescue medication. The use of other treatments, such as injections of any kind, moxibustion, acupuncture, or cupping, will not be allowed.

When combined with other diseases, the drugs and treatments must be continued, and the drugs and treatments need to be recorded in detail in the combined medication list.

#### Control group

One hundred patients in the control group will receive External Diclofenac Diethylamine Emulgel (Beijing Novartis Pharma Ltd. producing, trade name: Vitalin Emulgel) treatment (3–4 times per day for 10 weeks). Other interventions will be similar to the experimental group.

### Trial conduct

The personnel participating in the clinical trial shall accept the coincident training and then conduct the clinical trial after passing the examination. The radiologists and doctors will accept the training in filmmaking and reading. All MRI examinations and measurements are completed by Guang’anmen Hospital, Chinese Academy of Traditional Chinese medicine. The main investigator refers to the drug administrator who specifically supervises the study. The drug administrator shall keep records of drug receipt, distribution, and return. The quality inspectors will monitor the drug tablet return and laboratory tests, then write the audit report and submit it to the project team leader every month. The project management group is responsible for checking the research quality and progress of the subcenter every 3 months. Modifications to the protocol will be submitted to the Ethics Committee of Guang’anmen Hospital to review, and audits will be held annually by the Ethics Committee. The above process will be independent from investigators and the sponsor.

### Outcomes

The efficacy between the two groups will be assessed by primary outcome measure: change in WOMAC score three times (week 0, week 5, week10) (Fig. 1). The secondary outcomes measured at three time points (week 0, week 5, week10) included the Chinese Scale for Knee Osteoarthritis (CSKO), and at two points (week 0, week10) included *X-ray and MRI*.

In order to observe the time to effect, we measure the primary and secondary outcomes at week 5 and week 10, respectively; however, only the clinical results of week 10 were used as the final evaluation outcomes.

#### Primary outcome

##### The Western Ontario and McMaster Universities Osteoarthritis Index (WOMAC)

WOMAC score was performed for each follow-up, with the calculation of the total score of WOMAC, and each dimension was compared before and after treatment [21]. Clinical recovery: WOMAC score reduction by 95%; excellent: WOMAC score reduction by 70%; efficient: WOMAC score reduction by 30%; inefficient: WOMAC score reduction less than 30%. Note: the calculation formula [(WOMAC score before treatment − WOMAC score after treatment)/WOMAC score before treatment] × 100%.

#### Secondary outcomes

##### CSKO score

The Chinese Scale for Knee Osteoarthritis (CSKO) score was performed for each follow-up [22], with the calculation of the total score of CSKO being compared before and after treatment.

### X-ray data acquisition

Taking X-ray of the double knee joint with weight loading is at the adem position. The Kellgren and Lawrence classification criteria will be used on AP film to evaluate the degree of degeneration and the width of medial and lateral tibiofemoral joint space [17]. At the end of treatment, the X-ray of the double knee joint was taken again. Compare the width changes of medial and lateral tibiofemoral joint space on standard load positive X-ray before and after treatment.

### MRI data acquisition

Using Siemens 3.0T MRI to obtain the patient’s knee T2 mapping sequence. Using the Recht criteria to evaluate cartilage degeneration [23]. Measure the average thickness and volume of cartilage at the same time.

After the end of treatment, MRI images were taken of the affected limb for the MRI score of cartilage defect.

### Safety outcomes

(1) Vital signs: body temperature, resting heart rate, respiratory, and blood pressure after 10 min of rest (systolic blood pressure, diastolic blood pressure). (2) Laboratory examination: blood routine (RBC, WBC, platelet, hemoglobin), urine routines (urine red cell, LEU, urinary protein, glycated hemoglobin alc), stool routine+occult blood, and liver function (ALT, AST), and renal function (BUN, Cr, 12 lead routine electrocardiogram). (3) Manipulation-related adverse events, such as ecchymosis, subcutaneous hemorrhage, fracture, and dislocation. (4) Drug-related adverse events, such as gastrointestinal tract response. (5) Other adverse events.

The safety of each therapy was evaluated by comparing the changes in blood and urine routine, liver and kidney function, electrocardiography before and after the treatment, and the incidence of adverse events during the treatment.

### Data management and monitoring

The outcome assessors will use the CRFs to collect the data which consists of treatment effects, adverse events, and safety evaluations. Then, two data administrators who do not belong to the research team and are blinded to group allocation will independently receive the completed CRF and enter it into the Excel database (Microsoft, Redmond, WA, USA). They must complete rigorous data monitoring training. Then, they enter the real-time data into the China Clinical Trial Registry (http://www.chictr.org.cn), where the electronic data management system will be used for real-time tracking and monitoring of test data from the Research Office of Guang’anmen Hospital.

### Statistical analysis

All statistical analyses will be performed with the SPSS software (SPSS 20.0, SPSS Inc., Chicago, IL, USA) by statisticians who are independent of the research team and blinded to the group allocation. Data analysis will be based on the intention-to-treat (ITT) principles. All numerical data will be presented as the mean ± SD, and categorical variables will be described with percentages (%). All the statistical tests are bilateral inspection; *p* < 0.05 is considered statistically significant. Paired *t* test or the paired rank sum test is used for the comparison within the groups; the *χ*^2^ test or Fisher’s exact probability method is used for the count data, and the Wilcoxon rank sum test is used for the rank data between groups. CMH *χ*^2^ test is used for the primary outcome of WOMAC improve rate. It is necessary to adjust the baseline and centers of the changes in WOMAC score between the groups with the covariance method.

The secondary outcome includes CSKO score, X-ray data, and MRI data, which are all measurement data, and a group *t*-test or the Wilcoxon rank-sum test is used for comparison between the groups. The *χ*^2^ test or Fisher’s exact probability method is used for participants’ dropout and adverse events rate analysis.

### Ethics and dissemination

The protocol was designed following the principles of the Helsinki Declaration. Participants will be informed of the study protocol, possible risks, and other related matters before entering the study and sign the informed consent before randomization.

All original medical record test results (personal data, test documents) will be confidential within the law. The results of the trial will be shown in tables and figures only, and no individual will be identified. All data collected from this study can only be used for this research. All members of the research team have ethical principles of confidentiality.

The results will be published in peer-reviewed academic journals and disseminated through conferences.

## Discussion

Massage treatments through pressing, pinching, flexion-extension, stretching, plucking, rubbing, and other bone-setting manipulations are all recommended. According to traditional Chinese medicine (TCM), massage on the epidermis of the body can stimulate and adjust the distribution of meridians, qi, and blood. According to modern medicine, massage may improve systemic immune and inflammatory profiles in healthy individuals [24]. As a supplementary and alternative therapy, massage has been widely used in clinical practice and was widely recommended by Chinese orthopedic experts.

Although some studies shown effectiveness and safety for all patients with KOA treatment by traditional Chinese massage or Diclofenac solution, however, as far as we know, our study will be the first randomized controlled and multicenter clinical trial. In this study, evidence-based traditional Chinese medicine massage treatment VS External Diclofenac Diethylamine Emulgel will be conducted in participants with KOA to observe the correlation between clinical manifestations and changes in X-ray and MRI, so as to further understand and identify the effectiveness and safety of both treatment methods.

In order to avoid the bias of results and improve the reliability of clinical results, we try to keep the baseline consistency as much as possible. Participants will be screened strictly according to the inclusion and exclusion criteria. All the researchers will be trained to understand the design of the study. To achieve blindness in the course of the intervention, the sealed envelopes should be attached to massage and External Diclofenac Diethylamine Emulgel before the intervention. In order to maintain the consistency of massage techniques, all Chinese massage therapists involved in the study have achieved strict massage training. In the study, MRI images of the affected limb were taken for MRI score of cartilage defects in both groups. All participants will be carried out in the above five hospitals. This study also has several limitations. First of all, Chinese massage therapists and participants in the waiting list will not be blinded due to the nature of the intervention. Secondly, the long-term efficacy of massage therapy will not be observed due to the observation treatment period of the study is only 10 weeks.

## Trial status

The study protocol was approved by the Ethical Committee of Guang’anmen Hospital on 08 January 2018. The study is currently in the recruitment phase, and the first participant was included on 1 June 2018. We predict that recruitment will be completed by September 2021.

## Data Availability

The full protocol for the study will be available from the corresponding author. Datasets generated or analyzed during the current study will not be publicly available due to data privacy. Results from the trial will be published in peer-reviewed international journals, and positive, negative, and inconclusive results will be published.

## Abbreviations

KOA: Knee osteoarthritis
ACR: American College of Rheumatology
TCM: Traditional Chinese medicine
NSAIDs: Non-steroidal anti-inflammatory drugs
CRF: Case report form
WOMAC: Western Ontario and McMaster Universities Osteoarthritis Index
CSKO: Chinese scale for knee osteoarthritis
MRI: Magnetic resonance imaging
